# Intrinsic interactive reinforcement learning – Using error-related potentials for real world human-robot interaction

**DOI:** 10.1038/s41598-017-17682-7

**Published:** 2017-12-14

**Authors:** Su Kyoung Kim, Elsa Andrea Kirchner, Arne Stefes, Frank Kirchner

**Affiliations:** 1 Robotics Innovation Center, German Research Center for Artificial Intelligence (DFKI) GmbH, Bremen, Germany; 2 Robotics Lab, Faculty of Mathematics and Computer Science, University of Bremen, Bremen, Germany

## Abstract

Reinforcement learning (RL) enables robots to learn its optimal behavioral strategy in dynamic environments based on feedback. Explicit human feedback during robot RL is advantageous, since an explicit reward function can be easily adapted. However, it is very demanding and tiresome for a human to continuously and explicitly generate feedback. Therefore, the development of implicit approaches is of high relevance. In this paper, we used an error-related potential (ErrP), an event-related activity in the human electroencephalogram (EEG), as an intrinsically generated implicit feedback (rewards) for RL. Initially we validated our approach with seven subjects in a simulated robot learning scenario. ErrPs were detected online in single trial with a balanced accuracy (bACC) of 91%, which was sufficient to learn to recognize gestures and the correct mapping between human gestures and robot actions in parallel. Finally, we validated our approach in a real robot scenario, in which seven subjects freely chose gestures and the real robot correctly learned the mapping between gestures and actions (ErrP detection (90% bACC)). In this paper, we demonstrated that intrinsically generated EEG-based human feedback in RL can successfully be used to implicitly improve gesture-based robot control during human-robot interaction. We call our approach intrinsic interactive RL.

## Introduction

Reinforcement learning (RL) in real-world robotic applications is challenging for different reasons: a) the high-dimensional continuous state and action space, b) high-costs of generating real-world data (e.g., rollouts) and expensive real-world experiences which cannot be replaced by learning in simulation, and c) no straightforward way to specify appropriate reward functions including reward shaping to specify goals, etc^1–3^. These problems scale exponentially with the complexity of the task and the many pitfalls of the real world, which make it oftentimes impossible to decide whether or not an action was successful or failed.

Several approaches have been suggested to avoid specifying reward functions such as *inverse RL* ^4–6^, which extracts reward functions from demonstrations of the human expert, e.g., obtained by kinesthetic teaching or teleoperation, or *interactive RL* ^7–9^, in which the robot communicates with a human to improve robot’s behavior and learning speed. Recent approaches have focused on a more active contribution of the human to overcome the limitations of the initial approaches, especially of *inverse RL* (e.g., the so-called value alignment problem^10^). For example, in *cooperative inverse RL* ^11^, the human teaches the robot about the human’s reward function and robot and human together try to maximize the reward. Another example is *active reward learning* ^12^, in which the reward function is actively learned from the human as an expert while learning the policy.

In fact, the use of human feedback is advantageous in real-world robotic applications for different reasons. First, not all robotic applications allow us to define perfect reward functions because reliable ground truth measures of the robot’s actions are not available, e.g., gripping an object can be validated based on touch sensor data. However, the stability of the grip may not easily be derived from that data depending on the type of sensor used. Second, reward functions in real-world tasks are mostly hand-coded and require extensive task knowledge, which is not always available or faulty. Third, a reward function that was defined for a specific task *A* must be re-defined for task *B* even if *A* and *B* differ just slightly. However, human feedback can be obtained irrespective of task types or variation. Fourth, feedback in RL is based on a predefined reward function and usually discrete, and given for one specific action. Human feedback can more easily cover and hence validate a sequence of actions or even a subjective impression of behavior. Subjective correctness of behavior often is not a matter of a discrete decision and hence a subject which is difficult to be expressed and externalized even for a human. Thus, psychological measures, such as brain activity, are a good source of implicit feedback about complex internal evaluations made by a human observer that are hard to describe or to externalize. This is for example also known from the Uncanny Valley effect^13^ where the human may feel that a robot’s appearance or behavior is strange without being able to explicitly saying what is strange. However, this evaluation is clearly derivable from brain activity, e.g., by functional magnetic resonance imaging as shown for mismatches between the appearance of an agent and its motion^14^.

To make use of human feedback is of special interest in scenarios, in which the robot is directly interacting with a human^15^. Not only since the human is present anyway but also because during human-robot interaction the subjective sense of correctness might be more relevant than formal correctness. We could already show in several different robotic applications that the human electroencephalogram (EEG) encodes internal states, which can be detected online in single trial, by embedded brain reading^16–18^ and can be used to improve robotic behavior, e.g., smoother interaction, in rehabilitation tasks^19^ and user workload adjustments^20,21^.

In this study, we use intrinsically generated human feedback in a variant of interactive RL to improve human-robot interaction. We want to emphasize that we used human feedback as the exclusive reward source in contrast to most applications of interactive RL, in which human feedback is used in addition besides more conventional rewards generated by a predefined reward function^8^. We use *intrinsic human feedback*, i.e., a brain pattern called error-related potential (ErrP) as an implicit measure of the human evaluation of correctness of the robot’s actions.

The ErrP is an established event-related potential (ERP) component, which has been investigated in different application areas (for review^22^). It is elicited depending on task situation and therefore different types of ErrP can be specified, e.g., *interaction ErrP* ^23,24^, which is evoked by recognizing an error during interaction between human and machine, *feedback ErrP* ^25,26^, which is elicited by recognizing an error that is made aware by feedback presented to the human, *observation ErrP* ^24,27,28^, which is evoked, while observing an erroneous action of the robot (another person/external system, etc.), or *response ErrP* ^29,30^, which is elicited by recognizing the own error of the person who is performing a task that requires rapid responses (e.g., choice reaction task). Recently, ErrPs elicited by *execution* or *outcome* errors have also been reported^31^.

It has been investigated whether it is feasible to use ErrPs in single trial to evaluate the correctness of system behavior^24,28,32,33^, or to improve gesture recognition^34,35^. ErrPs have also been used to build a model in reinforcement learning tasks^32,36^. Further, ErrPs have been applied to robotic tasks to improve system performance using reinforcement learning^32,37–39^. For adaptive control of real robots, however, it is necessary to test the feasibility of the usage of ErrPs as online feedback not only while *observing* the robot’s actions^28,33,37,39^ but also during *interaction* with robots as suggested here in our study. In most previous studies (e.g.^33^), an explicit information about the correctness of the robot’s actions (ground truth) was displayed to the human to enable evaluation of the correctness of the robot’s actions while the human was observing the robot’s actions. This explicit information was necessary to detect ErrPs which were evoked while evaluating the correctness of the robot’s actions. Hence, the ground truth of the robot’s actions was predefined and this predefined ground truth was presented to the human while the robot was online correcting his/her actions based on ErrP detection^33^. In a recent experiment, however, it was enough that the subject knew the intended target position to elicit the ErrP, for the robot to learn an optimal strategy^39^. In other protocols outside the application field of human-robot interaction, subjects were even allowed to freely choose a movement target location that was not cued^38,40^. In our approach, the human performed freely-chosen gestures to communicate with the robot and the robot learns an action strategy online to perform correct actions according to human gestures. This kind of interaction between human and robot is beneficial, since the ground truth of the correctness of the robot’s actions can be implicitly generated in the human through an interaction with the robot via gestures. Thus, it is not necessary to display an explicit information about the correctness of robot’s actions to the human, since the human implicitly knows the correctness of the robot’s actions because the human decided on a mapping between gesture and robot behavior beforehand. This matches natural interaction conditions in which the mapping between command and response is not always predetermined or may change over time or differ between users. We developed experimental scenarios to test this kind of human-robot interaction/collaboration, in which ErrPs were used as the outcome of an evaluation that is delivered to the learning robot as feedback. Based on this feedback in RL, the robot implicitly learns the meaning of human gestures by online learning of the assignment between human gestures and the corresponding actions of the robot.

In summary, this paper proposes to use EEG as the only source of online feedback (reward) in RL tasks during human-robot interaction/collaboration. We use intrinsically evoked brain activities that do neither distract nor cause additional effort (externalization) on the human part. In our application, the robot learns the assignment of human gestures to corresponding actions and at the same time the recognition of human gestures using RL with ErrPs as rewards. A main contribution of this paper therefore lies in the efficient use of the human as a valuable critic in reinforcement learning robots. We make use of the unique intrinsic cognitive abilities of the human brain to evaluate observed complex behavior, while the human is actively communicating with the robot. This allows the robot to learn human gestures implicitly by means of ErrP-based RL. The approach was validated in a simulated as well as a real robot scenario and the applicability of intrinsically evoked human feedback (ErrP) in human-robot interaction/collaboration tasks could be demonstrated successfully. We consider our approach as a promising application of embedded brain reading in robotics.

## Methods

### Approaches

**Figure 1:**
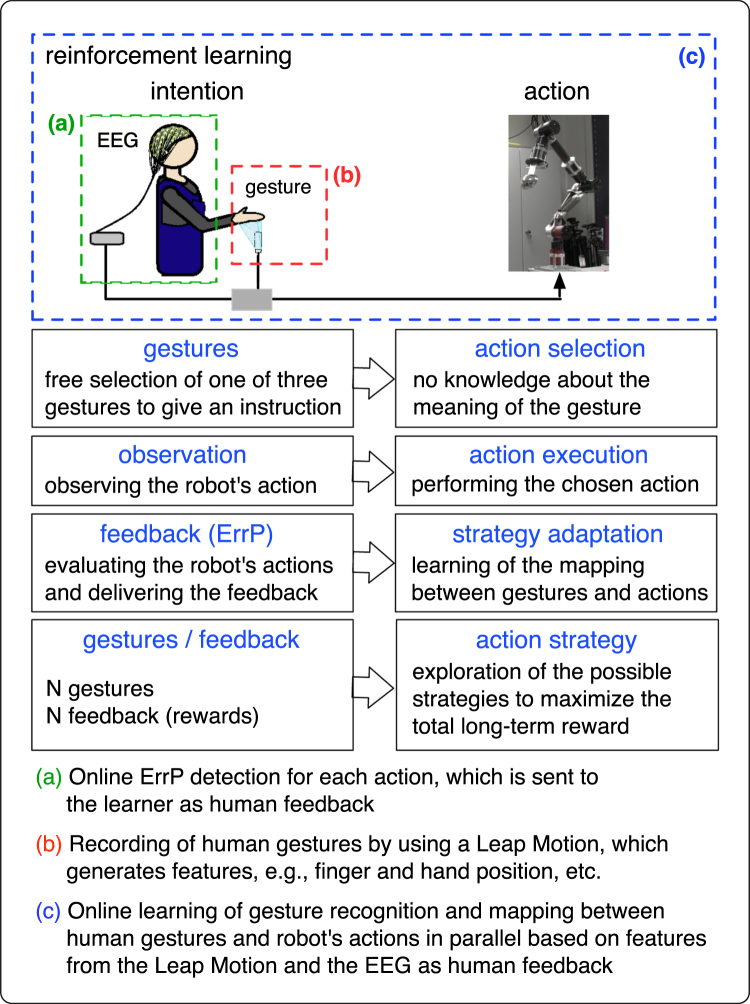
Concept of the proposed approach. The robot tries to find an optimal action strategy through interaction with the human. The robot explores the possible action strategies and receives feedback (rewards) from the human. The goal of the robot is to maximize the total reward in the long run. In this way, the robot can learn and adapt its action strategy, while the human freely chooses the gestures and delivers feedback to the robot. In the end, the robot implicitly learns the meaning of human gestures.

Figure 1 shows the schematic overview of the concept of the proposed approach (see also Supplementary Movie S1). We developed a human-robot interface, which enables the control of a robot by using human gestures. In our approach, the robot has no prior knowledge about the gestures before the robot receives feedback by interacting with the human. The robot learns the meaning of the gestures in a more indirect way by learning the assignment of human gestures to the corresponding actions. Here, we do not use a two-step procedure. The robot learns to recognize the human gestures based on gesture features extracted from a Leap Motion Controller^41^. In parallel, the robot learns the mapping between human gestures and robot actions by acting and receiving human feedback. On this account, theoretically, the human can change the meaning of the gestures, while the robot is learning the mapping between human gestures and robot actions. That means, relearning of gestures is possible.

For the learning of the mapping between human gestures and robot actions, we used a contextual bandit approach^42^, which enables to choose the robot’s actions based on context provided by human gestures. In our application, the user executes different gestures to control the robot and the robot chooses the actions depending on the gesture type. When the chosen action from the algorithm corresponds to the performed gesture of the user, the algorithm receives a positive feedback from the user. Otherwise, the user delivers a negative feedback to the algorithm. In this way, the algorithm learns a good policy for choosing actions based on a given context. That means, the action-selection strategy is updated with every action based on the feedback received from the user. To maximize the correct selection of actions in the long-term, the algorithm exploits the previous experiences and explores to gather new knowledge. Here, we tried to assure a robust learning through the stronger weighting of positive feedback compared to negative feedback.

As feedback (rewards), we used surface EEG signals measured from the user. When the user is recognizing a wrong mapping between the user’s gestures and the corresponding actions of the robot, an ErrP is evoked in the user, which is detected in real time and transferred to the learning algorithm as a negative feedback. In contrast, the algorithm receives a positive feedback, i.e., NoErrP, when the mapping is correct. Note that we defined that the positive feedback results in a higher absolute reward value (*r*_*t*_ = 1) than the negative feedback results (*r*_*t*_ = 0). This non-externalized kind of human feedback is a very effective way to communicate with the learner, since by the evaluation on the robot’s behavior brain activity (ErrP) is intrinsically evoked and detected for implicit feedback.

### Scenario Description

**Figure 2:**
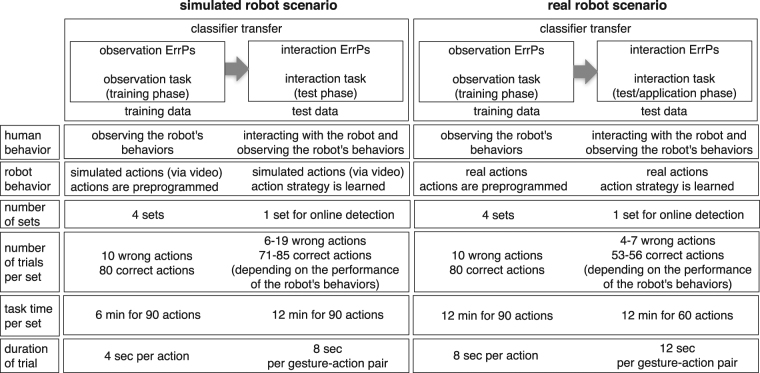
Scenario concept and task procedures. Each scenario contains a *training* phase to train a classifier and a *test* phase to evaluate this trained classifier. The reason for such classifier transfer is to reduce the calibration time. In the *training* phase, the subject observes the robot’s actions without interacting with the robot, i.e., without performing gestures (*observation* task). In this way, the time of data collection was substantially reduced in the *training* phase compared to the *test* phase that required an interaction with the robot (*interaction* task) by using gestures. In other words, we used the classifier trained on *observation ErrPs* to online detect *interaction ErrPs* in the *test* phase.

Figure 2 shows the schematic overview of the scenarios. We developed a simulated and real robot scenario to validate our approach. Both simulated and real robot scenario contain a training and test phase. In the training phase, the subjects did not interact with the robot. Instead, the subjects observed the robot’s actions without performing gestures (observation task). In contrast, in the test phase, the subjects interacted with the robot by using gestures (interaction task). Since the time to record the data took longer compared to the observation task, we used a classifier trained in the observation task to online detect ErrPs in the interaction task (classifier transfer approach). In previous studies, we could already show that calibration time can be reduced by applying such classifier transfer^24,43^. Both, the simulated and the real robot scenario followed the same concept for the training phase, i.e., the human performed no gestures. However, the test phase differed between simulated and real robot scenario. In the simulated robot scenario, subjects performed the gestures according to instructions. Hence, we could log all relevant data, i.e., *action instruction*, *action made by the robot*, *errors committed by the robot*, and *decision from a ErrP classifier*. In contrast, in the real robot scenario, the subjects could freely choose a gesture (no action instruction). Hence, we recorded a video of both gestures performed by the user and actions executed by the robot to evaluate ErrP detection performance and the robot’s performance.

#### Simulated Robot Scenario

**Figure 3:**
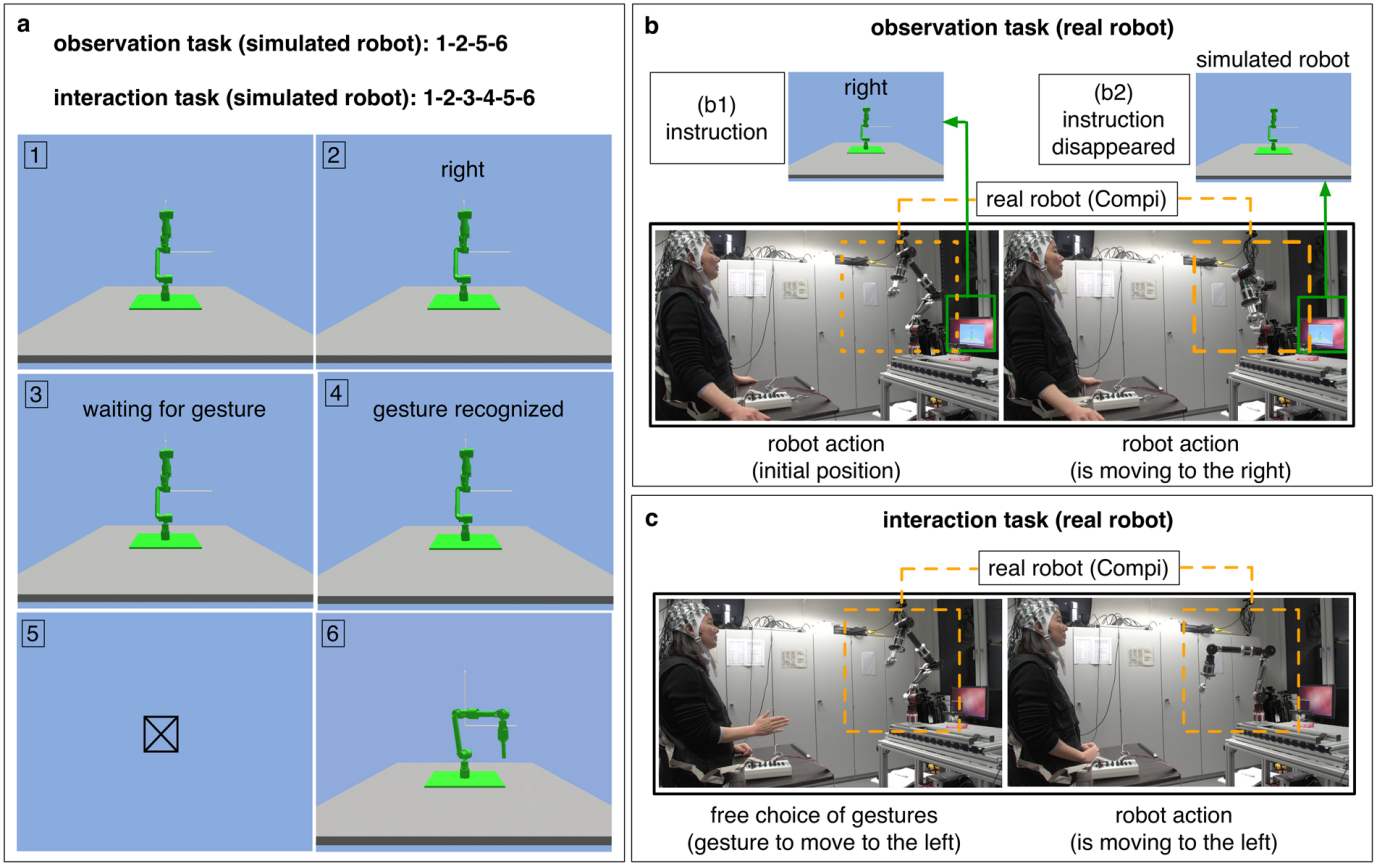
Simulated and real robot scenario. **(a)** Simulated robot scenario: In the observation task (training phase), four pictures were presented to the subjects: (1) the initial position of the robot, (2) the instruction for the robot, (5) the fixation cross, and (6) the action of the simulated robot. When the subjects recognized wrong actions of the robot, observation ErrPs were evoked. In the interaction task (test phase), two pictures were additionally presented to the subjects: (3) the message that requests gesture execution and (4) the conformation message that indicates that the gesture was successfully recorded. When the subjects recognized erroneous actions of the robot, interaction ErrPs were evoked. **(b)** Real robot scenario (training phase): The subjects observed the actions of the real robot. The instruction for the robot’s actions was presented on a monitor (b1) and disappeared after 1 s (b2). The real robot executed the actions that were preprogrammed. Observation ErrPs were evoked when the subjects recognized wrong actions of the real robot. **(c)** Real robot scenario (test phase): The subjects interacted with the robot by using gestures. When the subjects recognized wrong actions of the real robot, interaction ErrPs were evoked. In contrast to the simulated robot scenario, the subjects could freely choose the gestures to control the real robot.

In the observation task (training phase), subjects were instructed to only observe commands given by gestures and the robot’s actions. The experimental procedure is depicted in Fig. 3a. A total of three pictures and one video were presented to the subject in each trial (Figs 3a–1, 2, 5, 6). In the first picture, the initial position of the robot was presented to the subjects for 1 s. In the second picture, the instruction for robot control was presented to the subjects (e.g., please move the robot to the right). However, the subjects were not required to perform the corresponding gesture. Instead, gestures and the robot’s actions were preprogrammed. There was one video for each movement trajectory (action), i.e., forward, right and left movement of the robot, that were kept identical for each action type, since in the real robot scenario trajectories that the robot performed were also identical (pre-programmed) for each action type starting at the same position. Erroneous actions were simulated with a probability of 11%. After the instruction, a fixation cross was presented for 1 s. In the end, the executed action of the simulated robot was displayed to the subjects for 1 s as a video. Here, the robot was simulated by using the simulation tool MARS^44^. Note that the actions of the robot were not simulated online, but recorded beforehand. An observation ErrP was expected to be evoked in the EEG of the subjects, when the subjects observed and recognized an erroneous action of the robot.

**Figure 4:**
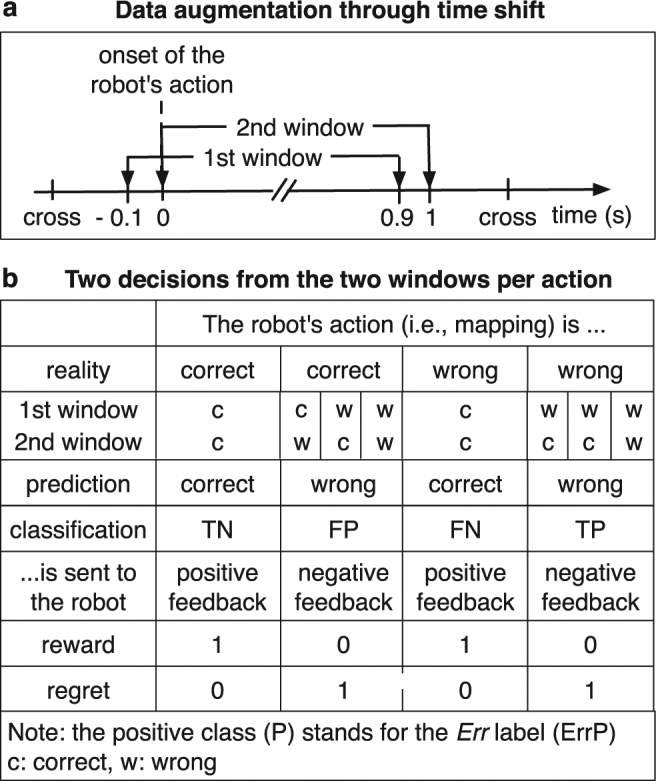
Data Augmentation approach. (**a**) Approach to find the interest of window for feature selection during continuous actions of the robot and (**b**) approach to handle few real-world data in robotic RL.

In the interaction task (test phase), subjects were instructed to control the simulated robot using a gesture recording system called Leap Motion^41^. We used three kinds of hand gestures to move the robot to the left, right or forward (Supplementary Fig. S1). The subjects moved their right hand to the left to move the robot to the left, they moved their left hand to the right to move the robot to the right, and they made a fist to move the robot forward (it was allowed to use either the right or the left fists here). The experimental procedure is depicted in Fig. 3a. A total of five pictures and one video were presented to the subject in each trial. With the first picture, the initial position of the robot was presented to the subjects for 1 s. By the second picture, the instruction for robot control was presented to the subjects (e.g., please move the robot to the right). Then, the subjects were required to perform the gesture, which corresponded to the previous instruction for robot control. The subjects had 10 s to perform the gesture. The picture did not disappear until the subjects performed the gesture. Here, it was allowed to skip a gesture, when the subjects were not entirely sure which gesture had to be performed, for example, the subjects missed the instruction for robot control. The next instruction was presented when the subjects performed no gestures for 10 s. In this case, the entire event was not included for evaluation. In this way, wrong gestures of the subjects were avoided. After performing a gesture, the subjects received the confirmation message that the gesture was successfully recorded. Note that the gesture performed by the subject (Figs 3a–4) was not yet recognized in our RL algorithm. Instead, the gesture was recorded and gesture features were extracted using Leap Motion^41^ at that moment. However we displayed “gesture recognized” to the subject, since this is more comprehensible. This message was displayed for 1 s. Afterwards, a fixation cross was presented for 1 s. In the end, the executed action of the simulated robot was displayed to the subjects for 1 s as a video, which was embedded in a custom presenter. As in the observation task, the robot was simulated by using the simulation tool MARS^44^. While the subjects controlled the robot, we measured EEG signals from the subjects. In cases in which a gesture of a subject did not assign to the intended robot action and the subject detected such mismatch (i.e., the erroneous interaction between the subject and the robot), we expected an interaction ErrP. Here, the online detection of the interaction ErrP (at the single-trial level) enabled us to automatically generate feedback for RL.

#### Real Robot Scenario

As in the simulated robot scenario, we used a training and test phase. The concept of the training phase (observation task) is the same as of the simulated robot scenario. The experimental procedure is depicted in Fig. 3b (see also Supplementary Movie S2). The instruction for robot control was displayed on the monitor for 1 s (Figs 3–b1). Afterwards, the instruction disappeared (Figs 3–b2) and the real robot began to execute the action. Subjects were instructed to observe the executed actions of the real robot. Executions of the robot’s action were differently long depending on the type of robot’s actions (left, right, forward) and took between 1.5 s and 2 s. As in the simulated robot scenario, robot’s actions were preprogrammed, in which erroneous actions were simulated with the probability of 11%. Thus, subjects did not need to perform gestures to control the robot. In the interaction task (test phase), subjects were instructed to freely choose gestures to control the real robot (Fig. 3c and Supplementary Movie S2). However, this free selection of gestures allows no ground truth to evaluate the performance of the robot. Hence, we recorded both gestures performed by the subjects and actions executed by the robot by video to obtain the ground truth for the robot’s performance. Again, we recorded EEG signals from all subjects. We detected online interaction ErrPs, when the subjects recognized the errors made by the robot.

For both training and test phase, two labels were generated for the classification: a) correct mapping between human gestures and robot’s actions (Corr), which leads to *no* occurrence of ErrPs (NoErrP) and b) wrong mapping between human gestures and robot’s actions (Err), which leads to occurrence of ErrPs (ErrP). The ratio of correct and wrong mapping was 1:8 for the training phase. However, in the test phase (both simulated and real robot scenario), the ratio of correct and wrong mapping was different depending on the performance of the robot’s behaviors (Fig. 2).

### Systems: gesture recording system, simulated and real robot

For gesture recording, we used a Leap Motion Controller (LMC)^41^. The LMC is a sensor, which connects with the computer via USB. The detection range is approximately 50 cm. The LMC has two monochromatic infrared cameras and three infrared LEDs. Each of both cameras records an image and using both cameras a stereo image is created. The LMC software determines the position of hand and finger bones in x, y, and z coordinates relative to the sensor. In the end, the LMC API provides the position and orientation of hand and fingers. The LMC API also allows to obtain a high-level information such as palm normal vector, direction, the posture of the hand (grab strength, pinch strength). For our application, we used the palm normal vector and grip strength as feature vectors, i.e., the x, y, z components of the palm normal vector and a value from zero to one, which describes how far the hand is opened or closed (from flat hand[0] to fist^1^). We recorded 10 samples (100 ms per sample) and generated a feature vector per sample. All feature vectors from 10 samples were averaged and this was used for the RL algorithm. Note that the RL algorithms received gesture features (raw values), but not the output of a separate gesture classification (i.e., recognized gestures) from the LMC API. For our application, we used three types of gestures: left, right, and forward gesture (Supplementary Fig. S1).

For the real robot scenario, we used a six degree of freedom (6-DOF) robotic arm called COMPI^45^ (Fig. 3b), which was developed at our institute (http://robotik.dfki-bremen.de/en/research/robot-systems.html). In this application, the robot arm was controlled by sending joint values over network to the robot control computer. Four predefined actions (left, right, forward, back to start) were implemented as a sequence of joint positions and three predefined actions (left, right, forward) were triggered from the learning system. For the simulated robot scenario, we simulated the robotic arm COMPI (Fig. 3a) by using the simulation tool MARS^44^ developed at our institute (http://robotik.dfki-bremen.de/en/research/softwaretools.html). The same approach as for the real robot control was used to control the simulated robot, but instead of network transfer, the joint values were sent. In the simulated robot scenario, we displayed the sequence of actions of the simulated robot to the subjects as a video, which was embedded in a custom presenter. That means, the actions of the robot were not simulated during the whole time of the task.

### Reinforcement learning (RL)

In our application, we used three kinds of gestures. That means, different types of actions should be chosen depending on gesture type. To this end, we used a contextual bandit approach^42^ as a variant of reinforcement learning, in which only one action is selected per episode. Here, a learning algorithm sequentially selects actions of the robot based on contextual information of the user’s gestures and robot’s action (i.e., assignment of the user’s gestures to the robot’s actions). The learner adapts the action-selection strategy based on feedback (ErrPs) received from the user. In the multi-armed bandit approach, context information is formulated as described in Li *et al*.^42^. The algorithm proceeds in discrete trials *t* = 1, 2, 3, …, *T*. For each trial, the algorithm observes the current user and a set ${{\mathscr{A}}}_{t}$ of arms together with the feature vector *x*_*t*_ per action. The vector *x*_*t*_ contains the context. Based on observed payoffs in previous trials, the algorithm chooses an arm *a*_*t*_ and receives payoff *r*_*t*_. The algorithm improves the arm-selection strategy with the new observation (*x*_*t*_, *r*_*t*_, *a*_*t*_). The total payoff of algorithm is defined as ${\sum }_{n\mathrm{=1}}^{T}{r}_{{x}_{t},{a}_{t}}$ and the optimal expected total payoff is defined as E [${\sum }_{n\mathrm{=1}}^{T}{r}_{{x}_{t},{a}_{t}^{\ast }}$]. To obtain the optimal expected total payoff, the expected total payoff should be maximized. In other words, the difference between the expected and the received total payoff $({R}_{A}(T)\,\mathop{=}\limits^{def}{\rm{E}}[{\sum }_{n\mathrm{=1}}^{T}{r}_{{x}_{t}}({a}_{t}^{\ast })]{\rm{E}}[{\sum }_{n\mathrm{=1}}^{T}{r}_{{x}_{t}}({a}_{t})])$ should be minimized. To minimize the regret, the algorithm exploits the previous experience to choose the best action. However, the algorithm has only a limited knowledge from the previous experience and thus the action-selection strategy is not perfect. For this reason, the algorithm explores to gather further knowledge to build the best action-selection strategy^46^. However, this is not limited to maximize the *current* reward. That means, in principle, the exploration can increase short-term regret, but can reduce long-term regret. Hence, we need a good trade off between exploitation and exploration. As algorithm, we chose the LinUCB algorithm^42^ that assumes that the expected payoff of an action *a* is linear in its feature *x*_*t*_ (context) with unknown coefficient vector ${\theta }_{a}^{\ast }$. To obtain the optimal trade off between exploitation and exploration, the LinUCB algorithm^42^ uses an upper confidence bound (UCB) algorithm^46–48^. For each trial (*t*), the algorithm estimates the mean payoff of each action $({\hat{u}}_{t,a})$ and its confidence interval (*c*_*t*,*a*_) and selects the action, which has the highest UCB [*a*_*t*_ = *arg max*_*a*_ $({\hat{u}}_{t,a}+{c}_{t,a})$, see line 11 in algorithm 1]. As mentioned earlier, we used different types of gestures and each gesture can be assigned to a particular action of the robot. Thus, the gesture features provide context. However, the robot’s action provides no context. Hence, we modified the original LinUCB algorithm. The exploration parameter (*α*) was empirically set to 2. Details are included in the Supplementary Materials.

**Algorithm 1:**
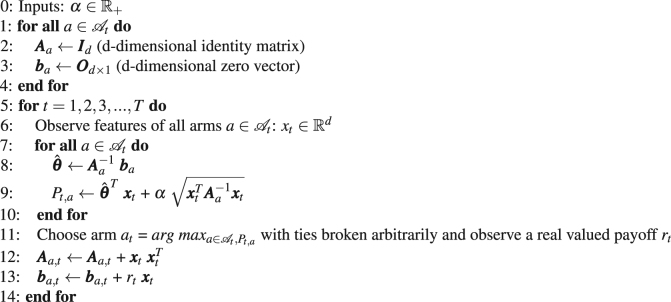
Modified LinUCB algorithm.

### EEG pattern as reward in RL

We used EEG pattern as feedback, i.e., we used positive and negative feedback provided by the classifier: 1 for correct mapping between human gestures and robot actions (*Corr*) and 0 for wrong mapping between human gestures and robot actions (*Err*): *r*_*t*_ = 1 or 0 in algorithm 1. As mentioned earlier, the collection of real-world data is high-costly and time-consuming in general and especially erroneous events do not often occur compared to non-erroneous events in real-world applications. Thus, it takes a long time to collect data containing ErrPs to train a classifier. To overcome this issue, we performed two approaches.

First, we augmented EEG data to receive two epochs (time windows which were used to extract features for the classifier) for the same event by a time shift during data segmentation (Fig. 4a). Hence, we received two decisions from the classifier for the same event (Fig. 4b). Only when we obtained the correct mapping from both time windows for the same event, a positive feedback, i.e., a feedback of *r*_*t*_ = 1 was sent to the learning algorithm. Otherwise, we send a feedback of *r*_*t*_ = 0 (Fig. 4b). That means, the positive feedback is more reliably obtained due to our data augmentation approach. Second, we emphasized non-erroneous events (correct mapping) that more often occurs in real-world experiences compared to wrong events (wrong mapping). Thus, the learning algorithm received 1 for a correct mapping and 0 for a wrong mapping. That means, positive feedback (*r*_*t*_ = 1) was updated (see, line 13 in algorithm 1) and had an effect on next action selection (see, line 8 and 9 in algorithm 1). In contrast, negative feedback (*r*_*t*_ = 0) was not updated (see, line 13 in algorithm 1). Nevertheless, negative feedback had also an impact on next action selection, since the features (context) was updated (see, line 12 in algorithm 1) and newly fitted (i.e., a new value of the estimated coefficient $\hat{\theta }$), which also affects next action selection (see, line 8 and 9 in algorithm 1).

In our application, positive feedback (*r*_*t*_ = 1) can be given in two cases (Fig. 4b): a) true negative (TN) classification (ErrP was *not* detected when the robot made *no* mistake) or b) false negative (FN) classification (ErrP was *not* detected although the robot *made* a mistake). Note that the positive class stands for a wrong mapping (*Err* label, ErrP). In contrast, negative feedback (*r*_*t*_ = 0) can be given in two cases (Fig. 4b): a) true positive (TP) classification (ErrP *was* detected when the robot *made* a mistake) or b) false positive (FP) classification (ErrP *was* detected although the robot made *no* mistake). To summarize, we tried to assure a robust learning by obtaining a higher reliability of positive events to overcome the under-supply of the real-world data in general and the rare occurrence of erroneous events.

For each subject, we pre-trained the algorithm by presenting the algorithm a gesture feature set (recorded from an additional subject) three times per gesture type as well as a simulated perfect ErrP based feedback to avoid the constant occurrence of wrong mapping in the early stage of learning (details in Supplementary text). In fact, in real-world applications usually learning does not always start at *zero*. Typically some knowledge is already available, e.g., some gestures are known but other might wanted to be added. However, sometimes training does start at *zero* also in real-world applications. Therefore, we additionally tested our approach in one subject (Subject 2) without pre-training. Online learning was found to be stable without pre-training. We obtained a balanced accuracy of 85% in the online ErrP detection (details in Supplementary text and Supplementary Fig. S2). Further, a similar pattern of regret was obtained with all subjects in the pre-training phase (Supplementary Fig. S3).

### Online ErrP detection

#### Subjects

Seven subjects (3 females, 4 males, age: 24.85 ± 7.4, right-handed, normal or corrected-to normal vision) participated in the simulated robot scenario study. In addition, nine subjects participated in the study using the real robot scenario. Two subjects from nine subjects were excluded: During the acquisition we had technical problems with the LMC loosing the signal caused by a loose USB cable connection for one of these two subjects. The cable was exchanged afterwards. One subjects moved too much during acquisition (in the break between the sets) such that the EEG cap moved backwards resulting in high impedances and big shifts in electrode positions. In the end, seven subjects (3 females, 4 males, age: 30.28 ± 8.3, right-handed, normal or corrected-to normal vision) were selected for the real robot scenario study. Two subjects (Subject 1 and Subject 2) participated in both simulated and real robot scenario.

All experiments were carried out in accordance with the approved guidelines. Experimental protocols were approved by the ethics committee of the University of Bremen. Written informed consent was obtained from all participants that volunteered to perform the experiments. Written informed consent for publication of identifying information/images was also obtained from all participants.

#### Data Acquisition

EEGs were continuously recorded using the actiCap system (Brain Products GmbH, Munich, Germany), in which 64 active electrodes were arranged in accordance to an extended 10–20 system with reference at FCz. Impedance was kept below 5 k Ω. EEG signals were sampled at 5 kHz, amplified by two 32 channel Brain Amp DC amplifiers (Brain Products GmbH, Munich, Germany), and filtered with a low cut-off of 0.1 Hz and high cut-off of 1 kHz.

#### Dataset

An overview of the dataset is illustrated in Fig. 2. For both scenarios, i.e., the simulated and real robot scenario, a total of five datasets was collected for each subject. Four datasets (training data) from the observation task were used to train a classifier and one dataset (test data) from the interaction task was used to evaluate the trained classifier. Hence, a classifier transfer (observation task → interaction task) was applied for both the simulated and the real robot scenario. For the training phase, each dataset contained 10 erroneous and 80 correct trials. For the test phase, online test data contained a different number of errors depending on the robot’s online performance (Fig. 2). This was caused by the difference in performance of the online applied learning algorithm, since its payoff is affected by the quality of feedback (i.e., the performance of online ErrP detection). For the simulated robot scenario, the task time per set took 6 minutes for collection time of training data and 12 minutes for the online test data. We needed more time for the online test data, since the subjects performed gestures in the online test. However, for the training data, the subjects did not perform gestures. Instead, they only observed the actions of the robot. For the real robot scenario, the duration of the real robot’s action took longer compared to the actions of the simulated robot. Thus, each set took 12 minutes for both the training and the online test data (Fig. 2).

#### Preprocessing

The EEG data was analyzed using a Python-based framework for preprocessing and classification^49^. The continuous EEG signal was segmented into epochs from −0.1 s to 1 s for each event type (correct/erroneous trial). Here, a challenge of online ErrP detection in our robot control scenario was to detect ErrPs without knowing when erroneous actions of the robot were recognized by the subjects. Another challenge is the variation of error recognition depending on the type of robot action (left, right, forward). That means, the onset of correct and erroneous events is unknown. Thus, we could not segment the EEG signals after each event type. Instead, we segmented the EEG signals after the start of the robot’s action. In other words, we began to detect ErrP after the onset of the robot’s action. The segmented correct trials did not overlap with the following erroneous trials, since a fixation point (cross) was presented for 1 s after each event type (Fig. 4a). That means, there was at least 1 s between the robot’s actions (i.e., between correct and erroneous events). Thus, only the correct trials without any error-related activity were labeled as correct. In the same way, only the erroneous trials without any correct-related activity were labeled as erroneous. All epochs were normalized to zero mean for each channel, decimated to 50 Hz, and band pass filtered (0.5 to 10 Hz). This procedure was also used in other studies^28^. The xDAWN spatial filter^50^ was used to enhance the signal-to-noise ratio. By applying the xDAWN the number of 64 physical channels was reduced to 8 pseudo channels.

#### Feature selection, feature extraction, and classification

Since we did not know the exact time point of the occurrence of the erroneous events (i.e., subjectively determined onset of the erroneous actions of the robot), we performed a pre-analysis to find an optimal window to detect ErrPs (details, Supplementary text and Supplementary Fig. S4). Based on this pre-analysis, we chose two time windows for feature extraction: [−0.1 s–0.6 s, 0 s–0.7 s]] for both simulated and real robot scenario. Features were extracted from eight pseudo channels after spatial filtering. We extracted a total of 280 features (8 pseudo channels ×35 data points =280 for each time window). Features were normalized over all trials and used to train a classifier. A linear support vector machine (SVM)^51^ was used to classify correct and erroneous trials. We optimized the cost parameter of the SVM (i.e., regularization constant^52^) and the class weight of underrepresented instances with a stratified five-fold cross validation using a grid search. We used the predetermined values [10^0^, 10^−1^, …, 10^−6^] for the cost parameter of the SVM and^1,2,4,6,8^ for the class weight of underrepresented instances. Note that we had an unbalanced ratio between erroneous and correct trials of 1:8. Hence, different penalty constants were used for two different classes^53^. As a metric for classification performance we used the arithmetic mean of true positive rate and true negative rate, balanced accuracy (bACC), where the erroneous trials belonged to the positive class.

## Results

### Online ErrP detection

In both simulated and real robot scenarios, ErrPs were elicited by erroneous behavior of the robot showing a characteristic waveform with fronto-central positive and negative peaks (Supplementary Fig. S5 and Supplementary text). Table 1 shows the online classification performance. Based on the number of trials the chance level should be around 58% and 60%^54^ for the simulated and real robot scenario respectively. For the simulated robot control, we achieved a high classification performance, (91% balanced accuracy (bACC) over all subjects). Further, we observed that there are variabilities between subjects (84–99% bACC). For the real robot scenario, we obtained a high classification performance as well (90% bACC over all subjects). Again, we observed variabilities between subjects (73–98% bACC). These very high performances in ErrP classification were achieved by our data augmentation approach. ErrP classification performance was improved for some subjects compared to a single window approach (Supplementary text and Supplementary Table S3).

**Table 1:** Online ErrP detection during a simulated and robot control (TPR: true positive rate, TNR: true negative rate, bACC: balanced accuracy [(TPR + TNR)/2]).

**Simulated robot scenario**.			
Training: observation task, Test: interaction task			
Subject	TPR	TNR	bACC
Subject 1 (female)	1.00	0.98	0.99
Subject 2 (male)	0.86	0.96	0.91
Subject 3 (female)	0.92	0.83	0.88
Subject 4 (male)	0.89	0.79	0.84
Subject 5 (male)	1.00	0.73	0.86
Subject 6 (male)	1.00	0.98	0.99
Subject 7 (female)	1.00	0.77	0.89
Mean ± SEM	0.95 ± 0.02	0.86 ± 0.04	0.91 ± 0.02
% CI	0.95 ± 0.06	0.86 ± 0.10	0.91 ± 0.06
**Real robot scenario**			
Subject	TPR	TNR	bACC
Subject 1 (female)	1.00	0.96	0.98
Subject 2 (male)	0.50	0.96	0.73
Subject 3 (female)	1.00	0.89	0.95
Subject 4 (male)	0.57	0.89	0.73
Subject 5 (male)	1.00	0.89	0.95
Subject 6 (female)	1.00	0.96	0.98
Subject 7 (male)	1.00	0.95	0.98
Average ± SEM	0.87 ± 0.09	0.93 ± 0.01	0.90 ± 0.04
% CI	0.87 ± 0.21	0.93 ± 0.03	0.90 ± 0.11

Mean, standard error of mean (SEM), and 95% confidence interval (CI = mean ± margin of errors are reported. Note that the positive class stands for a wrong mapping (*Err* label, ErrP).

### Performance of robot control

Table 2 shows the accuracy of robot actions for the simulated and the real robot scenario during total learning time. In both, we achieved a high performance.

**Table 2:** Performance of the simulated and real robot (accumulated number of wrong actions, total number of actions, and accuracy).

**Simulated robot scenario.l**			
Subject	Accumulated number of wrong actions	Total actions	Accuracy (%)
Subject 1 (female)	6	90	93.33
Subject 2 (male)	7	90	92.22
Subject 3 (female)	13	90	85.55
Subject 4 (male)	19	90	78.88
Subject 5 (male)	6	90	93.33
Subject 6 (male)	5	90	94.44
Subject 7 (female)	11	90	87.77
Mean ± SEM	9.57 ± 0.32	90	89.36 ± 3.40
% CI	9.57 ± 4.71	90	89.36 ± 5.23
**Real robot scenario**			
Subject 1 (female)	5	60	91.7
Subject 2 (male)	6	60	90.0
Subject 3 (female)	4	60	93.3
Subject 4 (male)	7	60	88.3
Subject 5 (male)	4	60	93.3
Subject 6 (female)	4	60	93.3
Subject 7 (male)	4	60	93.3
Mean ± SEM	4.86 ± 1.21	60	91.90 ± 2.02
% CI	4.86 ± 1.27	60	91.90 ± 2.12

Mean, standard error of mean (SEM), and 95% confidence interval (CI = mean ± margin of errors) are reported.

**Figure 5:**
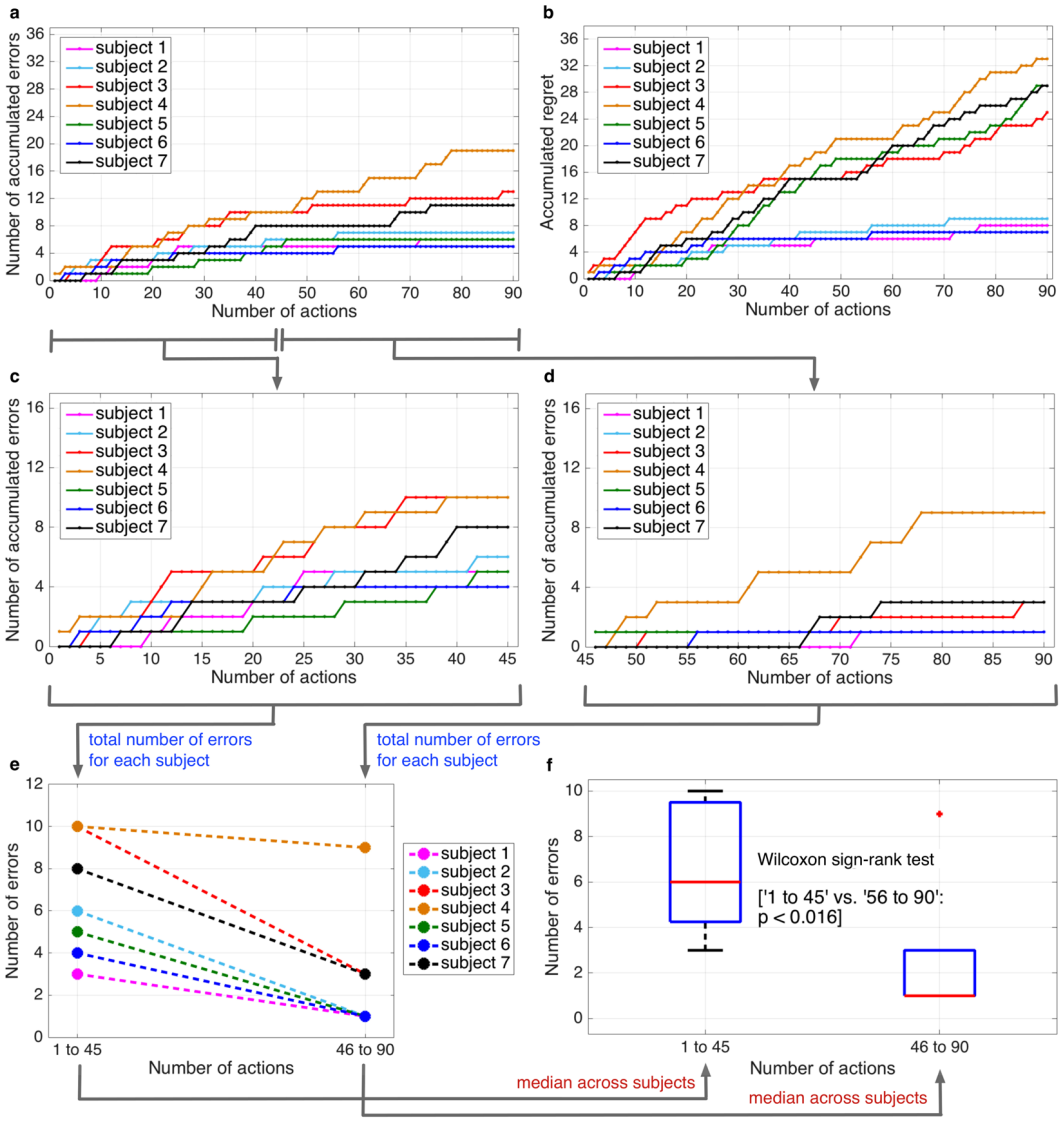
Simulated robot learning. (**a**) Accumulated errors of the robot for each subjects, (**b**) accumulated regret for each subjects, (**c**) accumulated errors of the robot in the first half of the experiment (1 to 45 actions) for each subject, (**d**) accumulated errors of the robot in the second half of the experiment (46 to 90 actions) for each subject, (**e**) total number of the robot’s errors in the first or the second half of the experiment for each subject, and (**f**) comparison between the first and last half of the experiment (1 to 45 actions vs. 46 to 90 actions) by performing Wilcoxon sign-rank test (two-sided, alpha = 0.05). The raw values (sample size of 7, i.e., 7 data pairs) used for this statistical analysis were depicted in (**e)**.

Figure 5a shows the accumulated errors of the simulated robot. In general, we observed a reduction of errors in the last third of the experiment. This pattern was shown for all subjects. The error curve was already stable in the middle of the experiment for all subjects except for one (Subject 4). For most subjects (Subject 1, Subject 2, Subject 5, and Subject 7), errors occurred more often at the beginning of the experiment compared to the end of an experiment. For Subject 3, we observed that the error curve stabilized very slowly. This subject also showed a higher total number of errors and more errors occurring in the beginning of the experiment compared to Subject 1, Subject 2, Subject 5, and Subject 7. However, we observed a stabilization of the error curve in the middle of the experiment. The highest total number of errors was obtained with Subject 4. For this subject, a very slow stabilization of the error rate was observed (the errors often occurred not only in the beginning but also still in the middle of the experiment).

Figure 5c and d show accumulated errors of the first and second half of the experiment. It can be seen that the amount of accumulated errors in the second half of the experiment was obviously smaller compared to the first half of the experiment. Figure 5e shows that the total number of errors for each subject was substantially higher for the first half of the experiment compared to the second half of the experiment. We observed that this tendency was not obviously shown for Subject 4. However, statistical evaluation (Fig. 5f) shows that the amount of accumulated errors was significantly reduced [Wilcoxon sign-rank test: first half of the experiment vs. second half of the experiment: *p* < 0.016, two-sided, alpha = 0.05]. Hence, error rate decreased over time by learning.

Not surprisingly, we observed a fast stabilization of error rate for subjects with high performance in online ErrP detection (Subject 1, Subject 2, Subject 5, Subject 6). In contrast, a slow stabilization of error rate was observed for subjects with lower performance in online ErrP detection (Subject 3, Subject 4 (Table 1 and Fig. 5a). Figure 5b shows the accumulated regret for each subject. We observed the correlation between the regret and the errors of the robot for all subjects except for one subject (Subject 5). For Subject 5, a small number of errors occurred, even though the regret was high. The reason for this is the high number of false positives in the online ErrP detection for this subject. In our approach, the false positives have less influence than false negatives (see Section Discussion). Thus, the learned model was stable despite of a relative higher number of false positives and thus a relative lower number of errors was shown for this subject. Otherwise, for all subjects showing a lower value of regret, less errors were also observed. In general, we observed that the higher the regret was, the more errors occurred.

**Figure 6:**
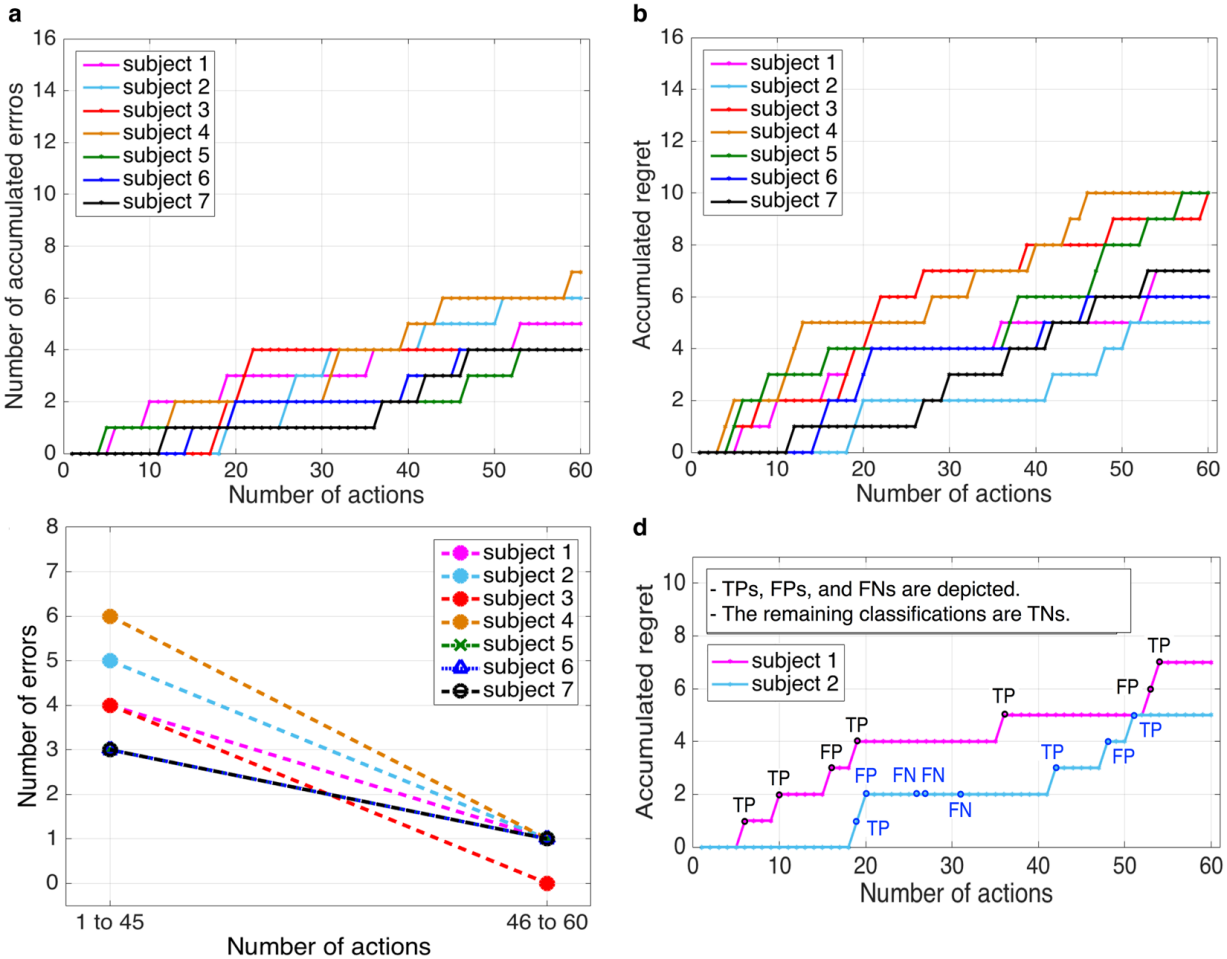
Real robot learning. (**a**) Accumulated errors of the robot for each subject, **(b)** accumulated regret for each subject, (**c**) total number of the robot’s errors over all subjects in the different phases of the experiment, (**d**) accumulated regret for Subject 1 and Subject 2.

Figure 6a shows the accumulated errors of the real robot. As expected, both the accumulated number of errors and the error curve were similar to the simulated robot scenario (Fig. 5a). Based on the results from the simulated robot scenario, which revealed that the number of errors of the robot’s action was significantly reduced after 45 actions, we grouped the action errors in the same way for the real robot scenario. This kind of grouping enables the comparison between the first part of both experiments (45 actions), i.e., the simulated and the real robot scenario. When comparing the first part of the experiment (with again actions 1 to 45) with the second part (here only 15 actions due to the shorter duration of the test run in the real robot scenario compared to the simulated robot scenario) again an improvement in behavioral performance, i.e., a reduction of errors, could be found (see Fig. 6c). However, one must be careful with the interpretation of this result, since the second half of the test experiment in the real robot scenario contained fewer actions. For this reason, we did not perform the statistical test as in the simulated robot scenario, but illustrated the results only in descriptive mode.

### Effect of ErrP detection performance on the robot’s behavioral performance

Our approach favours the true positive rate (TPR) compared to the true negative rate (TNR) (Fig. 4). Correspondingly, we found a correlation between the TPR and the robot’s performance [*r* = −0.899, *p* < 0.006] in the real robot scenario. Thus, the number of FNs had a stronger impact on the robot’s performance than the number of FPs (Supplementary Table S2). In fact, Subject 2 and Subject 4 who showed the worst accuracy of TPR achieved the worst performance in correctness of robot’s action compared to the remaining subjects in the real robot scenario.

Moreover, the robot’s performance was more affected when a high number of FNs and FPs occurred together compared to the occurrence of many FNs alone (Supplementary Table S2). The worst performance of the robot’s actions was achieved with Subject 4 and second worst performance was observed for Subject 3 who showed a large number of FNs and FPs in the simulated robot scenario (Supplementary Table S2). This finding is the reason why we found no correlation between the TRP and the robot’s performance in the simulation robot scenario. We found no large effect of the number of FPs alone except for Subject 7 (Supplementary Table S2). For this subject, gestures were poorly recognized (Supplementary text).

## Discussion

Our results show that EEG signals (ErrPs) can successfully be used as human feedback (rewards) in RL for learning in real-world robotic applications when a binary feedback is sufficient (binary reward specification). As expected, we observed that the higher the performance of online ErrP detection, the smaller the number of errors of the robot for most subjects. This result does not surprise, since high quality of feedback is the basis of efficient learning. In this context, a high accuracy of online ErrP detection in single-trials is relevant for online learning of action strategy of the robot. In fact, we could show a real-time ErrP classification with a high accuracy (91% balanced accuracy for the simulated robot scenario and and 92% balanced accuracy for the real robot scenario). Hence, the successive detection of ErrPs on the same task event, which was proposed in^33^ was not necessary in our study. In fact, these successive detections (due to high amounts of misclassification in the first robot’s actions) improved the classification performance in the case when a human observer recognized misclassifications of a ErrP classifier (e.g., ErrP was detected although the robot’s action was correct or ErrP was not detected although the robot’s action was wrong)^33^. However, the correction of the robot’s action by successively detecting ErrPs was possible only in binary tasks: the robot should pick and place the objects in the left *or* in the right. That means, the wrong actions of the robot (placing a object to the left) could be corrected (placing the object to the right) within a binary task^33^. Our approach is not limited to the number of actions due to the inherent property of RL.

Obviously, the regret curve did not exactly correspond to the performance of the robot’s actions (Fig. 5a vs. b, Fig. 6a vs. b), since the online ErrP detection was not 100% confident. In particular, in case of misclassification of wrong mappings (FN), i.e., the ErrP was not detected although the robot made a mistake, the learning algorithm, nevertheless, received a positive reward (*r*_*t*_ = 1) and updated the existing strategy for action selection accordingly. This was seen in Subject 2 (Fig. 6b): The regret was not increased when the ErrP was not detected although the robot made a mistake (misclassification of wrong mapping, FN). Note that the regret should be increased when the ErrP is correctly detected (correct classification of wrong mapping, TP). However, in most cases, we obtained correct classification of correct mapping (TN). The reason for the majority of TNs is that we double-checked the cases of TN by a data augmentation approach. In this context, more TNs can be generated than TPs (equivalently more FPs can be generated than FNs). This pattern can be seen in Fig. 5b. We observed higher accumulated values of regret for Subject 3, Subject 4, Subject 5, Subject 7. The reason for this observation is the higher number of FPs for these subjects. In fact, Subject 1, Subject 2, and Subject 6 had a lower number of FPs. Nevertheless, the learning of the mapping between human gestures and robot actions was in general not affected by the high number of FPs (Fig. 5b, Supplementary Fig. S2), since the learning algorithm updates the existing action strategy to a small extent according to the update of context (gesture features), but does not update based on the reward (in this case *r*_*t*_ = 0). In contrast, we obtained a higher reliability that the positive feedback (TN) provided by the classifier is surely correct. Furthermore, the obtained results indicate that our approaches to handle few real-world experiences in robotic RL (double-check of correct mapping through EEG data augmentation and more emphasis on correct mapping [positive feedback]) can be successfully applied to online learning of adaptive action strategies for robots. We stress again that our approach contributes to making less robot behavior errors, although the number of FPs is relatively higher. Such an approach does further help to handle situations in which the occurrence of an event in the EEG cannot be determined exactly as it is the case here. We do not know for sure at which time point after the robot started to perform an action the human observer recognized an error in its behavior. Such asynchronous behavior of ERPs in the EEG with respect to events must be handled with care. To consider any detection of ErrP as negative event and only repeated absences of ErrP (double NoErrP) as positive events does help to handle this issue of unknown ErrP onsets. Finally, for the real robot application we could clearly show that ErrP detection performance (i.e., TPR due to the reasons given above) has a clear influence on the robot’s behavioral performance.

As a first demonstration of our proposed approach, we have used a multi-arm bandit approach^42^. However, our approach does not allow to add further gestures on the basis of *the existing knowledge*, i.e., on the basis of the already learned gestures. Instead, in our approach, the learning of gestures can be completely relearned through interaction with a human, when further gestures should be added. In the present study, we have not tested how well the relearning of gestures is working in real applications. A systematic evaluation on this issue as well as the influence of performance (changes) in gesture recognition is needed in future work. Further, it is also interesting to investigate approaches that enable to add additional gesture-action mappings while retaining the already learned knowledge (i.e., retaining learned gesture-action mappings). In fact, which approaches are beneficial depends on real applications. When it is necessary to changes the meaning of the gestures due to new situations or applications, the relearning of gestures may be a good option. However, the learning of further gestures makes more sense, when the meaning of gestures should not be changed within the same application. Nevertheless, it may be useful to relearn human gestures when we consider that the generation of gesture features is not 100% perfect. In fact, this partly depends on the quality of the gesture recoding system as it can provide wrong features which strongly diverge from the gestures that were actually performed by the human (ground truth). A systematic investigation on this issue may be useful in future work.

Our study was designed such that the human directly communicates with the robot via gestures. The human implicitly provides the ground truth of the correctness of the robot’s actions. Hence, the human implicitly knows about the correctness of robot’s actions and it is not necessary to present the human an explicit information about the ground truth of the correctness of the robot’s actions. In principle, no guidance of the human is needed. The human can behave freely. However, in case of too many actions that a robot can perform, a pre-selection of possible actions by context of interaction or additional explicit input might be needed to avoid too long training of the RL approach in future work. Moreover, the expected negative effect on ErrP expression in case of an increased number of false behavior of the robot caused by e.g., many options available, and the effect of different levels of ErrP classification performance must still be investigated.

Furthermore, the development of approaches to enhance the benefit of using inherently generated human feedback (ErrPs) may be a relevant research topic in future work. The most important advantage of using the ErrP lies within its nature as an intrinsic, not externalized evaluation of a situation, which is done by the brain without the human being necessarily aware of it. This evaluation is the result of a complex analysis of a situation taking into account a rich set of experiences and a priori knowledge of the human observer. Therefore, this kind of feedback is most valuable in complex scenarios including many state/action pairs and even contextual information. An example could be a robotic system and a human working in a car production scenario to assemble the windshield into the car. Here the human is the experienced part that observes the doings of the unexperienced part (the robot) continuously and recognizes any suboptimal activity of the robot. These observations are not necessarily related to a very specific action in a specific state but are more likely an evaluation of a series of actions that the robot performed and that together resulted in a suboptimal performance. Even if the human does perceive the suboptimal performance immediately (and an ErrP is generated) there is no time for corrective statements of the human to the robot. Instead the intrinsically generated ErrP could be used as a feedback to the robot to improve its doing for the next windshield. Moreover, the feedback is instantaneous in its nature. There is in principle no need to wait for the robot to finish an action. Further, data processing with specifically optimized hardware^55^ can be performed within nanoseconds. How this very valuable feedback is used best in such a parallel continuous fashion is a question that must be studied further and can only be solved by means of adequate control architectures. Future work will therefore focus on questions such as scalability to an increased number of possible robot actions and continuous integration of ErrP based feedback. Using it directly could be an approach that would however require some form of background learning of the robot and foreground acting as it is known from a RL concept called *Dyna-Q* ^56,57^. ErrPs could also be used in an indirect way and be combined with an RL strategy that uses old experience for replay called *Experience Replay* ^57–61^.

In summary, we presented an intrinsic interactive RL approach using ErrP-based human feedback, which enables the learning of adaptive behaviors of a robot during interaction with a human. We showed that the assignment of freely chosen gestures to robot action can be learned by a robot during human-robot interaction based on specific intrinsically generated and online analyzed brain activity, i.e., brain states. That means, the robot does (in case of no pre-training) not know about the gestures at all in the beginning. Instead, the robot receives the input from gesture features from the gesture recording system. In case of pre-training, the robot has only few information on the gestures that might be chosen to control it. In addition, in both cases the meaning of the gestures is unknown to the robot and is learned by interaction. This kind of integration allows to relearn human gestures while learning to change gesture-action mapping online or to even adapt to new users with different gesture to action mappings online. Further, the real-time ErrP detection can be successfully used to send human intentions and evaluation on the robot’s behaviors to the robot. We achieved a high accuracy of the online ErrP detection for the simulated and real robot scenario (91% and 90%) although the onset of ErrP activity could not be determined beforehand, since for different users the subjective experience of error onset (in the robot’s behavior) may differ. We could also increase the reliability of successful online learning of adaptive action strategy of the robot by double-checking correct mappings using EEG data augmentation and by emphasizing correct mapping (positive feedback). In the end, we could demonstrate that the robot can adapt an optimal action strategy online by learning the mapping between human gestures (i.e. human intention) and its own actions based on ErrP-based RL. Since the brain pattern used as feedback is intrinsically generated by the human observer or interaction partner and needs no extra effort from the human this type of reinforcement learning can be called intrinsic interactive RL.

## Electronic supplementary material

Supplementary Information

Experimental procedure: Approach concept

Experimental procedure: Training and test phase
