# Supplementary material for: Intrinsic interactive reinforcement learning – Using error-related potentials for real world human-robot interaction
Source: Sci Rep. 2017 Dec 14;7:17562. doi: 10.1038/s41598-017-17682-7 (PMC5730605; doi:10.1038/s41598-017-17682-7)
Supplement: Supplementary file 1 — Supplementary Information [file 41598_2017_17682_MOESM1_ESM.pdf]

Supplementary Information

Intrinsic interactive reinforcement learning - Using error-related potentials for real world human-robot interaction

Su Kyoung Kim[1*], Elsa Andrea Kirchner[1,2], Arne Stefes[2], and Frank Kirchner[1,2]

[1]Robotics Innovation Center, German Research Center for Artificial Intelligence (DFKI) GmbH
[2]Robotics Lab, University of Bremen, Bremen, Germany
[*] Corresponding author: Su Kyoung Kim (E-mail: su-kyoung.kim@dfki.de)

This PDF file includes:
- Supplementary text
- Fig. S1: Gestures
- Figure S2: Pre-training vs. *no* pre-training
- Figure S3: Pre-training and gesture calibration in the simulated and real robot learning
- Figure S4: Time window analysis for continuous ErrP detection
- Figure S5: Grand averaged ERPs
- Table S1: The number of correct mapping between gestures and the robot's actions in the pretraining phase
- Table S2: The relationship between the ErrP detection performance and the robot's behavior performance
- Table S3: ERP detection performance in case of using two windows compared to the use of a single time window
- References

Other Supplementary Materials include the following:
- Movie S1 (.mov format): Experimental procedure: Approach concept
- Movie S2 (.mov format): Experimental procedure: Training and test phase

Supplementary Text

Pre-evaluation to determine optimal exploration parameter

We evaluated several exploration parameters (α) to find the optimal exploration parameter, which can balance exploitation and exploration for our application. To this end, we used the ErrP detection performance from our previous studies [1, 2] as feedback: true positive rate (TPR) of 0.71 and true negative rate (TNR) of 0.87. Based on this data, we tested different exploration parameters.We used the $\alpha \in$ N, where N = [0.25, 0.5, 1, 2, 4] and chose the parameter, which led to the lowest regret. Note that we fixed the number of gesture types (left, right, forward). We found that the parameter of 2 is best suited for our case. However, this optimal parameter, which was determined based on our previous studies [1, 2], can be differed from the scenarios and applications in the present study. Therefore, we further tested the upper and lower values of TPR and TNR around the ErrP detection performance of our previous study: [TPR of 0.6, TNR of 0.8] and [TRP of 0.8, TNR of 0.95]. Again, we found the exploration parameter of 2 was suitable for these cases.

Pre-training

We pre-trained the algorithm to avoid the constant occurrence of wrong mappings in early learning stage. This led to a speed-up of learning. Pre-evaluation of the quality and stability in the generation of feature sets from the LMC showed that not only between subjects but also within subjects individual feature sets would change a lot. Therefore, we decided against pre-training on individual feature sets recorded from the LMC but recorded one example feature set for each of the 3 gesture types with one subject not included in this study. These three feature set examples (one for each gesture type) were used to perform the pre-training. We pre-trained the algorithm by presenting each feature set for each gesture type (three gesture types, see Supplementary Fig. S1) randomly 3 times. After each presentation of a feature set, the prediction result of the algorithm was compared with the known presented gesture type and perfect simulated ErrP feedback was given. That means, 9 ideal feedback examples in total (3 ideal ErrP feedbacks for each gesture type) were given to the learning algorithm. This does not mean that any of the gestures was indeed learned (see, Supplementary Tab. S1), but by this approach we kept a similar level of pre-training for each subject. Indeed, a similar pattern of regret was obtained with all subjects in the pre-training phase (Supplementary Fig. S3). However, since gesture behavior (the subject's hand size, etc.) differed between subjects, for some subjects the feature set for all or some gestures could (by means of this procedure) have been different compared to the feature set recorded (from the excluded subject) for pre-training. In such a case, the learning is biased by poor gesture recognition. This was the case for Subject 7 in the simulated robot scenario. Video analysis revealed that gesture recognition was poor resulting in low performance in the robot's performance although the ErrP detection was perfect. Furthermore, we additionally tested our approach in one subject (Subject 2) without pre-training. We obtained an accuracy of 85% in online ErrP detection and a similar pattern of error occurrence of the robot's actions. This indicates that online learning is also stable without pre-training (Supplementary Fig. S2). However, the learning speed was slightly increased in case of pre-training compared to the case without pre-training. Further, it is noticeable that at the end of the experiment without pre-training many FPs could be observed. The reason for this is not clear. This finding might result from the subject's exhaustion, since the set without pre-training was performed at the end of the experiment. Finally, the effect might be a mere product of chance, since we tested only one subject. In future, this finding should however be topic of deeper investigation asking for approaches of online adaptation of classifier [3].

Pre-analysis for feature selection

We evaluated training data (from the observation task) to find out which time points within an epoch provide relevant features for the classifier. This analysis was necessary, since we did not know the exact time point of the occurrence of the erroneous events (i.e., subjectively determined onset of the erroneous actions of the robot). Thus, a relevant issue is to find the time windows that contain ErrPs. Here, we segmented epochs every 0.5s with a length of 1s. In the

end, we obtained 20 time windows, which started at Ns and ended at N+1 s, where N = [0.5, 1, ... 9, 9.5]. We trained the classifier on the segmented data and evaluated it by using a stratified five-fold cross validation. We obtained one classification performance for each time window. Hence, training was performed on each individual time window separately and tested to evaluate predictability of each individual window. Supplementary Figure S4 shows the results of the analysis of the time window. The best classification performance was achieved with the time window starting at 0.5 s (94% bACC). Second best, the earliest time window [0–0.25 s] was optimal for ErrP detection (above 93% bACC). We observed that the performance was high at this earliest time window and decreased for windows starting at 0.25 s and later, while performance increased again at 0.5 s. After 0.7 s performance decreased again. In fact, the different types of robot actions (left, right, forward) led to a wide distribution of optimal time windows. That means, erroneous actions of the robot were subjectively recognized at different times after robot action onset depending on the action the robot executed. The described analysis was performed with the subject who firstly participated in the study (Subject 1). The optimal time window, which was determined based on this subject, was used for online evaluation for all subjects, i.e., for online detection of ErrPs and online learning of the mapping between the subject's gestures and the executed actions of the robot. The optimal time window for this subject was as follows: [-0.1 s–0.6 s, 0 s–0.7 s]. Based on this pre-analysis, we chose two time windows for feature extraction for both simulated and real robot scenario [-0.1 s–0.6 s, 0 s–0.7 s]. We assumed that the combined use of both time windows for feature extraction leads to a more confident prediction.

Effect of ErrP detection performance on the robot's performance

We computed the correlation between the ErrP detection performance and the robot's behavior performance to investigate the effect of ErrP detection performance on the robot's behavior performance. There was a correlation between the accuracy of correct detections of ErrPs (TPR) and the robot's performance [$r = -0.899$, $p < 0.006$] in the real robot scenario. Our approach emphasizes more the accuracy of correct detections of ErrP (TPR) compared to the accuracy of wrong detections of ErrP (TNR) (see Fig. 4). Thus, TPR has a stronger impact on the robot's performance compared to TNR (see Supplementary Tab. S2). In this context, the number of FNs can strongly affect the robot's performance compared to the number of FPs. In fact, we also found a correlation between the number of FNs and the robot's performance [$r = 0.924$, $p < 0.003$] in the real robot scenario. In general, FPs occur more often than FN, and FP alone has no clear effect on the robot's performance due to our approach (see Fig. 4). However, when a large number of FNs occurs together with a large number of FPs, the robot's performance was more highly affected compared to FNs occurring alone (Subject 3 and Subject 4 in the simulated robot scenario, see Supplementary Tab. S2). A high number of FPs alone has no large impact on the robot's performance (see Supplementary Tab. S2) due to our approach (see Fig. 4). One exception was observed for Subject 7 in the simulated robot scenario (see Supplementary Tab. S2). The reason lies in the fact that 7 gestures were not correctly generated from the gesture features (revealed from video analysis). While ErrP detection worked perfectly in these examples the behavior of the robot was of course wrong (wrong gesture recognition resulted in wrong robot behavior).

Analysis of event-related potentials (ERPs)

As shown in Supplementary Figure S5, ErrPs were elicited by erroneous behavior of the robot showing a characteristic waveform with a positive peak between 332 ms and a negative peak around 540 ms in the real robot scenario. The positive and negative peaks were delayed in the simulated robot scenario, i.e., a positive peak around 504 ms and a negative peak around 584 ms. To compare the ERPs between the simulated and real robot scenario, we performed Wilcoxen sign-rank test (sample size of 7: seven comparison pairs from seven subjects). The statistical analysis shows that there was no significant difference in amplitudes between the simulated and real robot scenario in the positive peaks [$p = n.s.$]. However, the latency of the positive peak was significantly delayed in the simulated robot scenario compared to the real robot scenario [$p < 0.015$]. In addition, we found an increased negative peak in the simulated robot scenario compared to the real robot scenario for the electrode FCz [$p < 0.032$]. For the other fronto-central electrodes (FC1, FC2), there was no significant difference in amplitudes between the simulated and

real robot scenario [$p = n.s.$]. Further, we found a delayed negative peak in the simulated robot scenario compared to the real robot scenario [$p < 0.016$]. The negativity in the real robot scenario was broader compared to the simulated robot scenario. It can be assumed that the onset of error recognition during the robot's actions varies more strongly between the subjects compared to the simulated robot scenario. In fact, the robot's movement was slower in the real robot scenario than the simulated scenario. That can also be a reason why the subjects might have detected the errors in the real robot's action earlier. In the simulated robot scenario, the simulated robot's movement was faster and it is possible that the subject detected the action errors almost at the end of the robot's action. We assume that the subjects recognized the errors of the robot's actions earlier during the real robot movements compared to the simulated robot movements. The results can be also supported by the classification performance of each time window (see Supplementary Fig. S4), in which the classification performance was substantially reduced after 800 ms compared to the simulated robot scenario.

ErrP classification performance in double or single time window

For the online detection of ErrPs, we used two time windows and *NoErrP* was sent to the learning algorithm only in case that both time windows delivered the decision "correct mapping" (see Fig. 4). Hence, our approach ensures more accuracy for TPR. We obtained a high performance of online ErrP detection (see Tab. 1). In an offline analysis, we investigated how our data augmentation approach (i.e., the use of two time windows) can improve the classification performance compared to the use of a single time window (either first or second window). To this end, we additionally computed the performance when we used the first or second time window. Supplementary Table S3 shows that the classification performance was substantially reduced for some subjects in both simulated and real robot scenario when using a single window. In general, the classification performance was increased when using our data augmentation approach. In particular, we observed that the performance could especially be improved for subjects who showed the worst classification performance when using a single window (Subject 3 and Subject 4) in the simulated robot scenario (see Supplementary Tab. S3). These subjects also showed the highest number of errors in the robot's actions (see Tab 2 and Supplementary Tab S2). The same pattern was also revealed in the real robot scenario (Subject 2 and Subject 4) except for Subject 3.

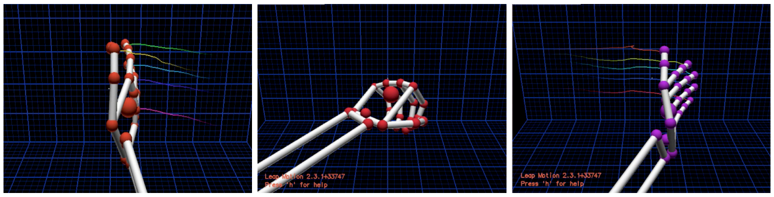

Figure S1: Gestures. We used three types of gestures (left, forward, right) to move the simulated and real robot scenario.

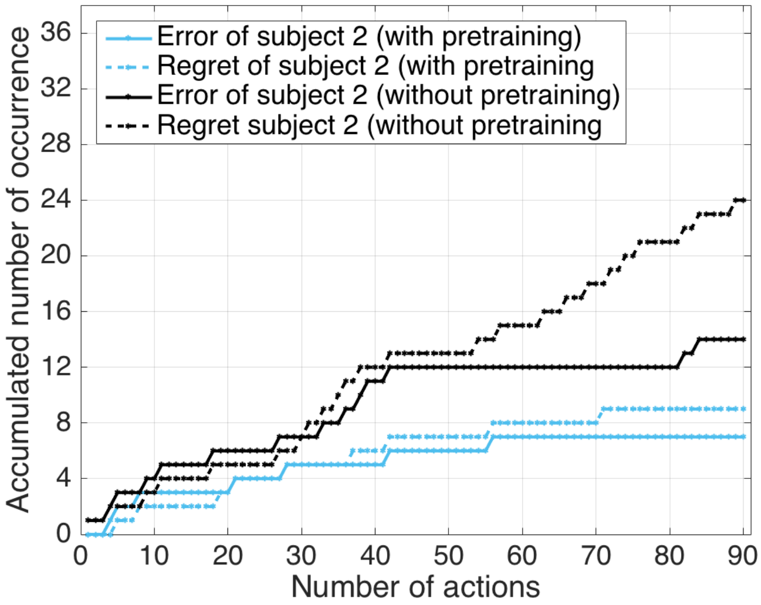

Figure S2: Pre-training vs. no pre-training in the robot learning. A similar pattern of error occurrence of the robot's actions was observed for the case of robot learning with pre-training and the case of robot learning without pre-training.

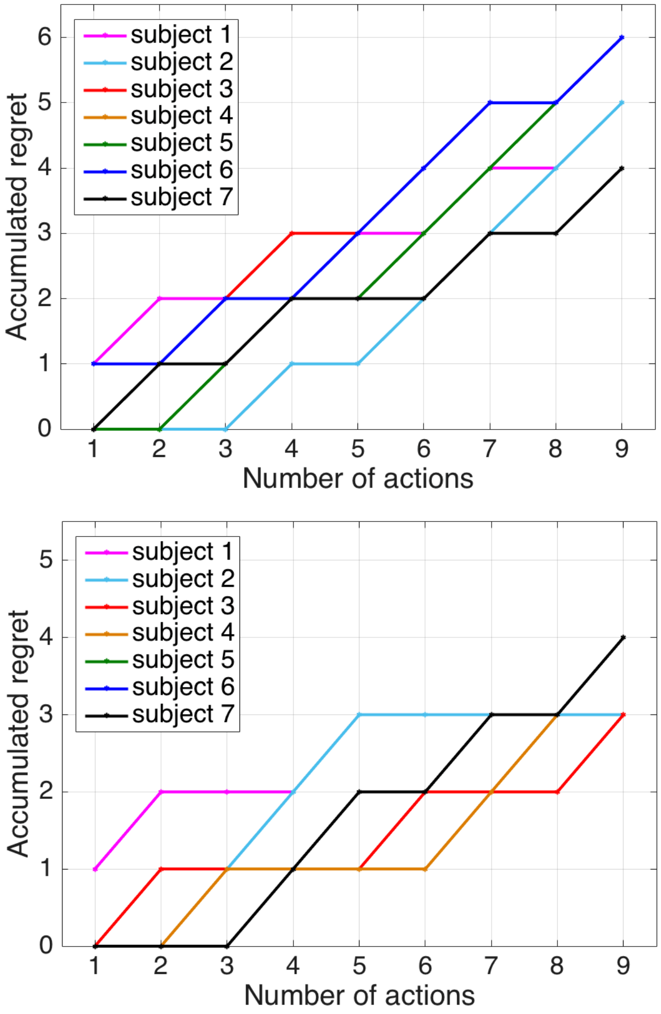

Figure S3: Pre-training in the simulated and real robot learning. (a) Pre-training in the simulated robot and **(b)** Pre-training in the real robot. A similar pattern of regret was obtained with all subjects in the pre-training phase.

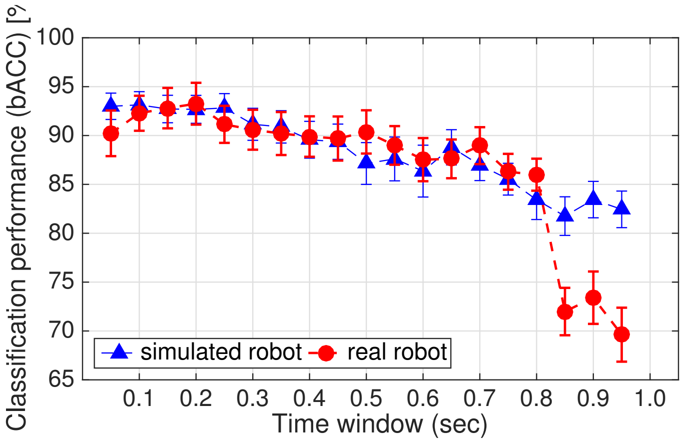

Figure S4: Analysis of time window. Classification performance (mean and standard error of mean) is depicted for each time window in Subject 1. Training data (observation task) from Subject 1 was analyzed offline to find out when the errors of the robot can be recognized by the human (i.e., the onset of ErrPs). To this end, the data of each time window was trained and tested (stratified five-fold cross validation). Based on this analysis, the optimal time window was selected for online ErrP detection and applied for all subjects (bACC: balanced accuracy [(TPR+TNR)/2].

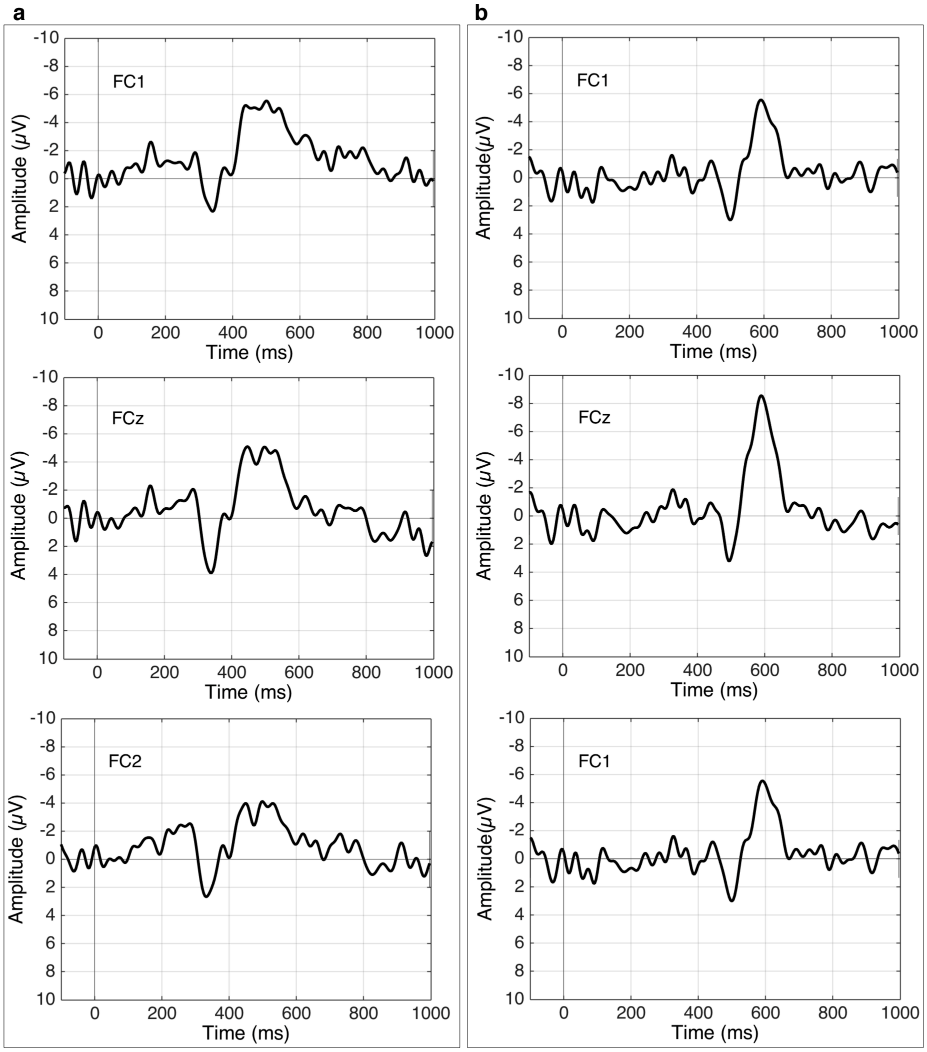

Figure S5: Grand averaged ERPs. **(a)** Grand averaged ERPs in channels FCz, FC1, and FC2 over all subjects (N = 7) in the real robot scenario. **(b)** Grand averaged ERPs in channels FCz, FC1, and FC2 over all subjects (N = 7) in the simulated robot scenario. Only artifacts-free trials are used. EEGs were filtered between 0.1 and $30\,Hz$. Difference between correct and wrong mappings are depicted.

Simulated robot scenario							
	Number of correct mappings			Robot performance	ErrP performance		
Subject	left	right	forward	number of wrong actions	TPR	TNR	bACC
Subject 1 (female)	1	2	2	6	1.00	0.96	0.99
Subject 2 (male)	2	2	1	7	0.75	0.95	0.91
Subject 3 (female)	3	2	1	13	1.00	0.89	0.88
Subject 4 (male)	3	2	1	19	0.57	0.89	0.84
Subject 5 (male)	3	2	1	6	1.00	0.89	0.86
Subject 6 (male)	3	2	1	5	1.00	0.96	0.99
Subject 7 (female)	1	2	1	11	1.00	0.95	0.89

Real robot scenario							
	Number of correct mappings			Robot performance	ErrP performance		
Subject	left	right	forward	number of wrong actions	TPR	TNR	bACC
Subject 1 (female)	0	1	2	5	1.00	0.98	0.98
Subject 2 (male)	3	2	1	6	0.50	0.96	0.73
Subject 3 (female)	3	0	0	4	0.92	0.83	0.95
Subject 4 (male)	2	1	1	7	0.89	0.79	0.73
Subject 5 (male)	3	2	1	4	1.00	0.73	0.95
Subject 6 (male)	1	2	0	4	1.00	0.98	0.98
Subject 7 (female)	3	2	0	4	1.00	0.77	0.98

Table S1: The number of correct mapping between gestures and the robot's actions in the pre-training phase and the accumulated number of the robot's wrong actions during the experiment and online ErrP detection performance. For the pre-training, the perfect feedback was given. (TP: true positive, TN: true negative, FP: false positive, FN: false negative, TPR: true positive rate, TNR: true negative rate, bACC: balanced accuracy [(TPR+TNR)/2]). Note that the positive class stands for a wrong mapping (*Err* label, ErrP).

Simulated robot scenario								
Subject	TP	TN	FP	FN	TPR	TNR	bACC	Number of the robot's errors
Subject 1 (female)	6	82	2	0	1.00	0.98	0.99	6
Subject 2 (male)	6	80	3	1	0.86	0.96	0.91	7
Subject 3 (female)	12	64	13	1	0.92	0.83	0.88	13
Subject 4 (male)	17	56	15	2	0.89	0.79	0.84	19
Subject 5 (male)	6	61	23	0	1.00	0.73	0.86	6
Subject 6 (male)	5	83	2	0	1.00	0.98	0.99	5
Subject 7 (female)	11	61	18	0	1.00	0.77	0.89	11

Real robot scenario								
Subject	TP	TN	FP	FN	TPR	TNR	bACC	Number of the robot's errors
Subject 1 (female)	5	53	2	0	1.00	0.96	0.98	5
Subject 2 (male)	3	52	2	3	0.50	0.96	0.73	6
Subject 3 (female)	4	50	6	0	1.00	0.89	0.95	4
Subject 4 (male)	4	47	6	3	0.57	0.89	0.73	7
Subject 5 (male)	4	50	6	0	1.00	0.89	0.95	4
Subject 6 (female)	4	54	2	0	1.00	0.96	0.98	4
Subject 7 (male)	4	53	3	0	1.00	0.95	0.98	4

Table S2: The relationship between the ErrP detection performance and the robot's behavior performance. The subjects were aligned (ranked) based on the ErrP detection performance, i.e., bACC (TP: true positive, TN: true negative, FP: false positive, FN: false negative, TPR: true positive rate, TNR: true negative rate, bACC: balanced accuracy [(TPR+TNR)/2]). Note that the positive class stands for a wrong mapping (*Err* label, ErrP).

Simulated robot scenario													
	(1) two windows			(2) first window			(3) second window			diff (1)-(2) [%]		diff (1)-(3) [%]	
Subject	TPR	TNR	bACC	TPR	TNR	bACC	TPR	TNR	bACC	TPR	bACC	TPR	bACC
Subject 1	1.00	0.98	0.99	1.00	0.98	0.99	0.83	0.98	0.91	0	0	17	8
Subject 2	0.86	0.96	0.91	0.86	0.96	0.91	0.67	0.96	0.82	19	9	0	0
**Subject 3**	0.92	0.83	0.88	0.86	0.83	0.85	0.75	0.82	0.79	**6**	**3**	**17**	**9**
**Subject 4**	0.89	0.79	0.84	0.89	0.79	0.84	0.65	0.76	0.71	**0**	**0**	**24**	**13**
Subject 5	1.00	0.73	0.86	0.85	0.73	0.79	1.00	0.73	0.86	15	7	0	0
Subject 6	1.00	0.98	0.99	1.00	0.98	0.99	1.00	0.98	0.99	0	0	0	0
Subject 7	1.00	0.77	0.89	1.00	0.77	0.89	0.92	0.77	0.85	0	0	8	4

	(1) two windows			(2) first window			(3) second window			diff (1)-(2) [%]		diff (1)-(3) [%]	
Subject	TPR	TNR	bACC	TPR	TNR	bACC	TPR	TNR	bACC	TPR	bACC	TPR	bACC
Subject 1	1.00	0.96	0.98	1.00	0.96	0.98	1.00	0.96	0.98	0	0	0	0
**Subject 2**	0.50	0.96	0.73	0.50	0.96	0.73	0.43	0.96	0.70	**0**	**0**	**7**	**3**
**Subject 3**	1.00	0.89	0.95	1.00	0.89	0.95	0.80	0.89	0.85	**0**	**0**	**20**	**10**
**Subject 4**	0.57	0.89	0.73	0.5	0.88	0.69	0.44	0.88	0.66	**7**	**4**	**13**	**7**
Subject 5	1.00	0.89	0.95	1.00	0.89	0.95	1.00	0.89	0.95	0	0	0	0
Subject 6	1.00	0.96	0.98	1.00	0.96	0.98	1.00	0.96	0.98	0	0	0	0
Subject 7	1.00	0.95	0.98	1.00	0.95	0.98	1.00	0.95	0.98	0	0	0	0

Table S3: ErrP detection performance in case of using two windows compared to the use of a single time window (first or second window). Note that the positive class stands for a wrong mapping [*Err* label, ErrP] (TPR: true positive rate, TNR: true negative rate, bACC: balanced accuracy [(TPR+TNR)/2]).

References

[1] Su Kyoung Kim and Elsa Andrea Kirchner. Classifier transferability in the detection of error related potentials from observation to interaction. In *Proceedings of the IEEE International Conference on Systems, Man, and Cybernetics, (SMC)*, pages 3360–3365, 2013.

[2] Su Kyoung Kim and Elsa Andrea Kirchner. Handling few training data: classifier transfer between different types of error-related potentials. *IEEE Transactions on Neural Systems and Rehabilitation Engineering*, 24(3):320–332, 2016.

[3] H. Woehrle, M. M. Krell, S. Straube, S. K. Kim, E. A. Kirchner, and F. Kirchner. An adaptive spatial filter for user-independent single trial detection of event-related potentials. *IEEE Transactions on Biomedical Engineering*, 62(7):1696–1705, July 2015.
